# A Cross-Sectional Study of Myopia and Morning Melatonin Status in Northern Irish Adolescent Children

**DOI:** 10.1155/2023/7961623

**Published:** 2023-11-01

**Authors:** Jane M. Fulton, Sarah C. Flanagan, Julie J. Sittlington, Diego Cobice, Sara Dobbin, Sara J. McCullough, Gareth Orr, Patrick Richardson, Kathryn J. Saunders

**Affiliations:** ^1^Centre for Optometry and Vision Science, Biomedical Science Research Institute, Ulster University, Coleraine, UK; ^2^Nutrition Innovation Centre for Food and Health (NICHE), Biomedical Science Research Institute, Ulster University, Coleraine, UK; ^3^Mass Spectrometry Centre, Biomedical Science Research Institute, Ulster University, Coleraine, UK

## Abstract

**Purpose:**

Previous studies have demonstrated an association between melatonin status and both refractive error and axial length in young adult myopes. This study aimed to determine if this relationship extends to a younger adolescent cohort.

**Methods:**

Healthy children aged 12–15 years provided morning saliva samples before attending Ulster University (55°N) for cycloplegic autorefraction and axial length measures. Participants completed questionnaires describing recent sleep habits and physical activity. Salivary melatonin was quantified using high-performance liquid chromatography-tandem mass spectrometry. Data collection for all participants occurred over a 1-week period (April 2021).

**Results:**

Seventy participants aged 14.3 (95% CI: 14.2—14.5) years were categorised by spherical equivalent refraction [SER] (range: −5.38DS to +1.88DS) into two groups; myopic SER ≤ −0.50DS (*n* = 22) or nonmyopic −0.50DS < SER ≤ +2.00DS (*n* = 48). Median morning salivary melatonin levels were 4.52 pg/ml (95% CI: 2.60–6.02) and 4.89 pg/ml (95% CI: 3.18–5.66) for myopic and nonmyopic subjects, respectively, and did not differ significantly between refractive groups (*P* = 0.91). Melatonin levels were not significantly correlated with SER, axial length, sleep, or activity scores (Spearman's rank, all *P* > 0.39). Higher levels of physical activity were associated with higher sleep quality (Spearman's rank, *ρ* = −0.28, *P* = 0.02).

**Conclusion:**

The present study found no significant relationship between morning salivary melatonin levels and refractive error or axial length in young adolescents. This contrasts with outcomes from a previous study of adults with comparable methodology, season of data collection, and geographical location. Prospective studies are needed to understand the discrepancies between adult and childhood findings and evaluate whether melatonin levels in childhood are indicative of an increased risk for future onset of myopia and/or faster axial growth trajectories and myopia progression in established myopes. Future work should opt for a comprehensive dim-light melatonin onset protocol to determine circadian phase.

## 1. Introduction

Significantly higher melatonin concentrations have been detected in young myopic adults compared to their nonmyopic peers [1]. This difference between refractive groups was investigated further in a distinct unrelated cohort of young adults by Flanagan et al. [2] using a dim-light melatonin onset protocol (DLMO). Marking the endogenous starting point of melatonin secretion under dim-light conditions, the DLMO is the gold-standard marker for assessing the circadian phase [3]. The authors found that while the myopic group's melatonin levels were consistently elevated throughout the study period compared to the nonmyopes, the time of DLMO did not differ between refractive groups, suggesting that elevated melatonin levels found in myopes could not be attributed to a dysregulation or delay of the circadian phase.

Study outcomes are not consistent across geographical locations. Findings in Northern Ireland [1, 2] have been corroborated in a cross-sectional study of Indian myopes [4] which reported higher morning serum melatonin levels in adult myopes than emmetropes. In contrast, investigations conducted in Texas, USA, by Abbot et al. revealed no difference in morning salivary melatonin levels [5], mean daily melatonin [6], or amplitude of diurnal variation [6] between myopic and emmetropic adults. In Australia, Chakraborty et al. found adult myopes to have a significant delay in the circadian phase compared to emmetropes as assessed by DLMO [7]. The delay in DLMO was positively correlated with the degree of myopia and reflected an objective delay in sleep onset in the myopic cohort. Chakraborty et al. also reported significantly lower overnight melatonin secretion in myopes compared to nonmyopes, assessed via urinary 6-sulfatoxymelatonin levels. Chakraborty et al. did not collect morning melatonin samples, thus limiting comparison to previous refractive studies. The differing methodologies and analytical techniques employed by each study have likely contributed to these inconsistent findings [8].

Rising levels of melatonin in the evening (during the DLMO phase) induce a state of quiet wakefulness which helps promote sleep [9]. Melatonin is therefore known as the “sleep hormone.” Studies investigating the relationship between sleep quality and duration and myopia in children and young adults also present conflicting outcomes. A study conducted by Ayaki et al. using the Pittsburgh Sleep Quality Index (PSQI) reported decreased quality and shorter sleep duration in highly myopic children aged 10 to 19 years [10]. Poorer sleep quality scores were significantly correlated with myopic refractive error. Significant differences were detected between high myopes and children with little or no myopia; however, no significant differences in sleep metrics were observed between children with little myopia or no myopia. Questionnaire/survey-based studies in Korea [11] and China [12] investigating children aged 12 to 19 years and six to 18 years, respectively, found shorter sleep durations to be associated with myopic refractive errors; however, this relationship was not identified in Australian children using direct measurement techniques [6, 13] nor in two other studies investigating myopia and self-reported sleep duration in Chinese children [14, 15].

Seasonal changes in behaviour, perhaps influenced by significant variations in photoperiod, light intensity, and light exposure patterns, also appear to influence sleep patterns [13] and circadian rhythm [16–18] in both myopic and nonmyopic children. Myopic children show more variability in sleep duration across days and seasons than nonmyopic children [13]. Furthermore, faster myopia progression and axial elongation have been reported throughout the winter season [19, 20]. Many studies exploring the link between sleep, melatonin, and refractive error have been conducted over extended periods which obfuscate underlying relations.

The aim of the present study was to determine whether the higher levels of melatonin previously reported by our laboratory in the morning saliva of adult myopes [2] are also evident in younger, adolescent individuals whose eyes are actively growing. Data collection occurred over a restricted time period to eliminate potential seasonal variations in melatonin concentration. Self-reported sleep and physical activity data were also collected.

## 2. Methods

### 2.1. Participants

This research was reviewed by an independent ethical review board (Ulster University Research Ethics Committee REC/18/0102) and adheres to the tenets of the Declaration of Helsinki. Each participant and their parent/guardian provided informed written assent and consent prior to participation in the study. Children who had taken part in the Northern Ireland Childhood Errors of Refraction study and consented to be recontacted were invited to participate along with age-appropriate siblings or friends. Data collection occurred over one week in April 2021 during school holidays. This study is reported following the Strengthening the Reporting of Observational Studies in Epidemiology (STROBE) guidelines (S2 STROBE Checklist).

### 2.2. Inclusion/Exclusion Criteria and Sample Size

Participants were children aged 12–15 years. With the exception of age, exclusion criteria were aligned with those applied by Flanagan et al. to provide a comparable cohort for analysis and enable screening of variables known to impact circadian rhythms or the interpretation of study outcomes. Exclusion criteria included (1) astigmatism greater than −2.00DC, (2) cycloplegic autorefraction greater than +2.00DS, (3) use of medications affecting melatonin levels (e.g., melatonin supplements and anti-inflammatory drugs), (4) history of eye disease and/or previous eye surgery, and (5) trans-meridian travel within the last month. None of the myopic participants were undergoing myopia management with optical or pharmacological interventions.

A sample size calculation using salivary melatonin data from a previous study of myopic and nonmyopic young adults [2] indicated 22 participants in each refractive group (power 85%; significance 5%). Given the at-home saliva sampling protocol, target sample sizes were inflated in an attempt to ensure a sufficient number of samples of suitable quality and volume for melatonin analysis that were obtained.

### 2.3. Sample Collection and Melatonin Analysis

Participants provided an at-home saliva sample on waking on the morning of data collection before attending the Ulster University campus (55°N) for the remainder of data collection. Home or “free-field” collection of saliva is a safe, easily understood, common, and well-recognised methodology used in research and clinical studies [21]. Home collection of saliva allowed participants to provide samples in a controlled environment upon waking without the need for an overnight stay in a sleep laboratory and was consistent with methodology utilised by Flanagan et al. [2].

A saliva collection kit was delivered to each participant. Participants were asked to watch a video demonstrating the gold-standard passive drool collection technique prior to the morning of data collection. Saliva collection instructions aligned with recommendations provided by Salimetrics LCC [22]. Twelve hours prior to providing their saliva sample, participants were asked to drink only water, stay well hydrated, avoid cigarette smoke, aspirin, and anti-inflammatory medications, and avoid eating bananas, chocolate, grapes, cereals, olives, and nuts [23, 24]. On the morning of data collection, participants were asked to refrain from turning on lights or opening curtains to ensure light levels remained as low as possible until saliva collection was complete. Upon waking, participants rinsed their mouths with water and minimised movement, remaining in a seated position. Participants were also asked to refrain from brushing their teeth, chewing gum, and using lip make-up. Ten minutes later, participants tilted their heads forward and let saliva pool on the floor of their mouths before drooling the sample through a SalivaBio Collection Aid (Salimetrics LCC, State College, PA, USA) and into a polypropylene vial. A minimum of 2 ml saliva was deposited. Participants stored their saliva samples in the refrigerator before attending for data collection later that day. Samples were collected from participants and stored at −80°C prior to analysis. Analysis of melatonin was by liquid/liquid extraction and high-performance liquid chromatography tandem mass spectrometry (HPLC-MS/MS).

High performance lipid chromatography was a Shimadzu Nexera XR (Kyoto, Japan), and separation was performed in the gradient mode. Mass spectrometer was an API 4000 triple quadrupole (QQQ) (AB Sciex, Warrington, UK) equipped with a turbo ion spray source. Melatonin powder, ≥98%, was purchased from Sigma-Aldrich (Dorset, UK). Melatonin-d4 (internal standard, ISTD) was purchased from Cambridge Bioscience (Cambridge, UK). All other reagents were from Sigma-Aldrich unless otherwise stated. The bioanalytical method detailed in the supporting information (S1 Appendix) was developed and validated in accordance with the International Council for Harmonisation (ICH) of Technical Requirements for Pharmaceuticals for Human Use guidelines for bioanalytical method validation [25] by the Mass Spectrometry Centre at Ulster University.

Participants were asked a series of questions on the day of the study visit to confirm they met the eligibility criteria and that the procedure for saliva collection had been followed correctly. Saliva samples were collected and analysed as described above.

### 2.4. Ocular Measurements

Cycloplegic drops (1 drop of 1% cyclopentolate hydrochloride) were instilled if a recent (within 3 months) cycloplegic refraction result was not available from participation in a previous research study. Thirty minutes following instillation, refractive error, axial length, and corneal curvature were measured using the Oculus Myopia Master (OCULUS, Germany) which was calibrated prior to use. Refractive error was recorded as spherical equivalent refractive error (SER).

Participants completed two self-report questionnaires prior to the study visit: the Pittsburgh Sleep Quality Index (PSQI) and the Physical Activity Questionnaire for Children (PAQ-C). Both questionnaires have been successfully administered in previous research studies involving child participants [26–29] and were used to explore general sleep behaviour and activity levels and the relationship between these parameters and melatonin, SER, and axial length. Given the age of participants, item 8 of the PSQI was modified to remove reference to driving [30].

Parents and guardians of participants were advised that they may assist their child in completing the questionnaires. This may have helped minimise any potential questionnaire-associated recall bias.

Myopic and nonmyopic participants were recruited from the same nonclinical population and were provided with identical instructions detailing saliva sample collection methodology and limiting the influence of information or selection bias on melatonin results. Authors had access to potentially identifying information during data collection.

### 2.5. Statistical Analysis

Statistical analyses were performed in SPSS (IBM SPSS Statistics for Windows Version 27.0). Cycloplegic SER did not differ significantly between right and left eyes (*P* > 0.99). Only right eye data are considered further. Age of participants, SER, axial length, time of saliva sample collection, bedtime, sleep latency, wake time, time asleep, global PSQI score, and PAQ-C score were examined to determine their relationship with morning salivary melatonin concentration. Participants were classified as myopic (mean SER ≤ −0.50DS) or nonmyopic (mean SER >−0.50DS to ≤+2.00DS) based on their right eye cycloplegic SER [31]. PSQI and PAQ-C questionnaires were scored according to their respective published guidelines, and global PSQI summary scores were utilised to label sleep quality as “good” or “poor” as recommended by Buysse [28]. Data were analysed for normality using the Shapiro–Wilk test. Age, SER, melatonin concentration, sleep, and activity scores were all non-normally distributed; therefore, Spearman's Rank Order Correlation was used to test for correlations between variables. The Mann–Whitney *U* test was used to explore differences between the groups. In all instances, a *P* value of <0.05 was considered statistically significant. Adult data from Flanagan et al. [2], whose protocols aligned closely with those of the present study, were compared directly with those from the adolescent participants in the present study.

## 3. Results

Seventy-eight 12–15-year-old children attended for data collection. Seven participants' data were incomplete and removed from analyses; three participants failed to provide saliva samples, three provided insufficient saliva samples for analysis, and one participant's data were excluded due to compromised data collection. Levels of melatonin detected ranged from 0.55 to 16.6 pg/ml. The highest melatonin concentration detected (16.6 pg/ml) was identified as an outlier by the Tukey method; this participant's data were removed from all analyses as this result was higher than expected for daytime saliva samples analysed by mass spectrometry [32–35]. The adjusted range was 0.55–13.3 pg/ml (*n* = 70). The remaining participants' data contained no missing variables.

Table 1 describes participant demographics, SER, axial length, morning salivary melatonin concentration, sleep, and activity scores by the refractive group. Median morning salivary melatonin levels were 4.52 pg/ml (95% CI: 2.60–6.02) and 4.89 pg/ml (95% CI: 3.18—5.66) for myopic and nonmyopic participants, respectively. Saliva sample collection time did not vary significantly between the groups (Mann–Whitney, *P* = 0.93). More females participated in the study than males (61%); however, no significant association between sex and melatonin level was detected (Mann–Whitney, *P* = 0.25).

**Figure 1:**
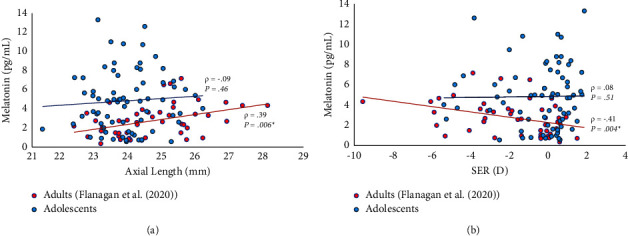
Melatonin concentration was not associated with (a) axial length or (b) SER in adolescent participants. Concentration of morning salivary melatonin was significantly related to (a) axial length and (b) SER in adult participants but not in adolescent participants of the present study. In both studies, morning saliva samples were collected at home, upon waking. One adolescent participant's melatonin level (16.6 pg/ml) was identified as an outlier and is not included in this figure. Adult data were provided by Flanagan et al. [2] *ρ* = Spearman's correlation coefficient. ^*∗*^Indicates significance at *P* < 0.05.

With the exception of the expected significant correlation between SER and axial length (*ρ* = −0.64, *P* < 0.001), no significant correlations were observed between melatonin and SER or axial length (Figure 1) or between melatonin, SER, axial length and PAQ-C score, global PSQI score, or individual PSQI components (Spearman's rank, *P* > 0.19 for all). Spearman's rank correlations of all variables with melatonin are detailed in Table 2.

As seen in Table 1, there were no statistically significant differences in self-reported physical activity levels between refractive groups (*P* = 0.47); however, children reporting higher levels of physical activity reported significantly better sleep quality as characterised by their global PSQI score (*ρ* = −0.28, *P* = 0.02).

Significant correlations were also observed between individual PSQI components. Adolescents sleeping for longer periods reported earlier bedtimes (*ρ* = −0.28, *P* = 0.02) and later wake times (*ρ* = 0.34, *P* = 0.004). Later bedtimes were also correlated with later wake-up times (*ρ* = −0.28, *P* = 0.02).

## 4. Discussion

This study explored the relationship between morning salivary melatonin concentration and refractive status in school-aged children for the first time. To constrain seasonal effects, all data were collected during a single week and the methodologies were aligned to allow comparison of outcomes with previous adult studies from our laboratory [2]. Saliva samples were analysed for melatonin using a highly specific mass spectrometry technique.

Melatonin concentrations in the present study ranged from 0.55 to 13.3 pg/ml with a median of 4.82 pg/ml (interquartile range 2.42 to 6.76 pg/ml). These levels are comparable to other studies using alternative, less precise measurement techniques to describe morning salivary melatonin concentration in children (median 6.76 pg/ml; interquartile range 5.98–8.64 pg/ml [36] and mean approximately 8.00 pg/ml [37]). The melatonin concentrations identified in the children participating in the present study were generally higher than those determined in young adults by the mass spectrometry technique reported by Flanagan et al. [2] (median 2.62 pg/ml; interquartile range 1.46–3.62 pg/ml). This comparison is illustrated in Figure 1. Children are known to have, on average, higher levels of melatonin than adults and the children in the present study would be expected to demonstrate a depletion in melatonin levels with increasing age [38]. Melatonin data from one participant was identified as an outlier. Study outcomes were not impacted following removal of this participant from analyses.

Despite collecting data at the same time of the year and geographical location as adult studies conducted by Kearney et al. [1] and Flanagan et al. [2] and using comparable methodology, the present study found no significant difference in melatonin concentration between myopic and nonmyopic children, and no significant relationship between melatonin and axial length or SER. The reason for the disparity demonstrated in the relationship between refractive status and melatonin status in adulthood compared to adolescence is not clear from the present data and cannot be resolved without prospective study. Neither do the current data allow us to determine whether melatonin status prior to the onset of myopia is related to future refractive status.

If melatonin plays a role in ocular growth and development, as indicated in animal studies [39–42], one could hypothesise that nonmyopic participants with relatively higher levels of morning melatonin in the present study are “premyopes” who will become myopic in the future. Prospective studies including younger cohorts would allow us to evaluate whether melatonin status influences the rate of eye growth and/or development of refractive error over time. If circadian rhythms influence refractive development during childhood, they may provide a mechanism with which to modify refractive outcomes in the future.

Alternatively, perhaps differences in circadian physiology between myopic and nonmyopic eyes identified in adults are a consequence of myopia and only become measurable or manifest at higher levels of myopia or with larger eye size. As expected from the younger participant profile, there was a smaller magnitude of myopia and eye size in the present study. The median myopic participant in the present study exhibited 1.02D less myopia and 0.80 mm shorter axial length than adult participants in a previous study [2]. Previous work has established that sleep metric differences between myopes and nonmyopes are only recorded when myopia reaches higher levels [10]. The retina of a longer, more prolate, or more myopic eye may be subject to altered light signalling and/or other challenges to the circadian system introduced by myopia. Another challenge when exploring the association between circadian physiology and refractive error and the effect of age on this relationship is the relatively wider spread and generally higher levels of melatonin found in adolescents which may mask interrefractive group differences in this cohort.

While the present study quantified levels of free, unbound plasma melatonin in morning salivary samples, other studies have examined biological fluids such as blood serum and urine [1, 2, 4, 7]. A recent study has demonstrated the potential for melatonin to be detected and quantified in human tears [43]. While we cannot make direct comparisons of “absolute” levels of melatonin between biological fluids, it would be interesting for future studies to determine whether differences in melatonin levels between myopes and nonmyopes can be detected in human tears.

Ideally, prospective studies should aim to characterise DLMO, rather than utilise single melatonin samples collected during daylight as the latter methodology is known to have limitations, particularly when considering the circadian phase [8, 44, 45]. Given the interplay between choroidal thickness and myopia [46] and the presence of ocular diurnal rhythms in both axial length and choroid thickness in the human eye [47, 48], the circadian phase may be more important in influencing eye growth and refractive development during childhood than the magnitude of circulating melatonin sampled at one time point. In the present study, we assessed melatonin levels at a single time point in the morning, and while this was useful to compare our adolescent data with that from previous studies using similar methodology, these values represent the relapsing phase of the circadian rhythm and cannot be extrapolated to indicate the circadian phase. Rather, a more challenging DLMO protocol is required to profile the rise of melatonin secretion in the evening and robustly compare the circadian phase between individuals and groups. Such a protocol was not within the resource or practical scope of the present study.

In agreement with previous adult studies within our laboratory [2], no relationship was detected between children's self-reported physical activity or sleep metrics and morning melatonin concentration. Neither were associations found between self-reported sleep quality (global PSQI score) and refractive error or axial length. These findings align with a previous study of children and young adults aged 10–19 years conducted by Ayaki et al. who evaluated the association between myopia and self-reported sleep quality identified through application of the PSQI questionnaire [10]. While the authors found myopia of −6.00D or more to be significantly correlated with “poor” PSQI sleep quality, short sleep duration, and late bedtimes, children with lower levels of myopia (which correspond more closely to the refractive status of participants in the present study) did not differ in terms of sleep quality, duration, or bedtime metrics when compared to nonmyopic children. None of the participants in the present study presented with −6.00D or more of myopia.

Children in the present study who were more physically active self-reported better sleep quality than those who were less active, but there was no relationship between physical activity and refractive status in the present study. Previous reports [49, 50], including a study conducted in the same underlying population as the present study [51], have reported that increased physical activity is associated with less myopia in childhood; however, no evidence of physical activity as a risk factor for myopia independent of time spent outdoors has been identified. It may be relevant to note that data collection occurred during Covid-19 restrictions where formal sports training was not permitted by the Northern Irish Executive [52] and 24% of participants felt that their usual physical activity had been disrupted as a consequence. Despite this disruption, the identified association between physical activity and sleep is consistent with outcomes of a recent meta-analytic review which found regular exercise to have a moderate beneficial effect on sleep quality [53].

The present study benefits from a tight data collection period, comprehensive inclusion/exclusion criteria, and robust methodology for detection and quantification of melatonin. All salivary samples were obtained over a one-week period, removing the potential confounding influence of season on melatonin concentration. Medical, ocular, and travel histories were obtained to ensure comprehensive screening criteria were met. Menstrual cycle was not considered in female participants as morning salivary samples were obtained, and while fluctuations in melatonin concentration with the menstrual cycle phase have been reported, particularly in night-time samples of melatonin, findings remain ambiguous [54–56]. Similarly, the onset of puberty could influence melatonin concentrations detected in this cohort of young adolescents, and this factor was not considered in analysis [57]. Standardised protocols for collection and handling of saliva for melatonin analysis were followed [58]. Unstimulated “gold-standard” passive drool saliva samples were obtained in a seated position, and levels of free melatonin were quantified by HPLC-MS/MS analysis. Mass spectrometric methods are highly specific to the target compound and show a decreased risk of cross contamination in comparison to other assay-based analytical techniques [32, 33, 59]. Saliva samples were stored in the fridge/freezer after collection and, while not specifically protected from light exposure, previous work reports no difference in the degradation of melatonin in samples stored in light compared to light-protected conditions [60].

The authors acknowledge that a lack of consideration for the circadian phase and variation in the rate of melatonin clearance by the liver may have resulted in high interindividual variation in morning melatonin concentration and obfuscated underlying associations between melatonin levels and myopia. Further limitations of this study include the use of self-report questionnaires as an indicator of prior sleep and physical activity and home collection of saliva samples. Questionnaires such as the PSQI used in this study are subject to questionnaire-associated recall bias and, due to the influence of social factors, tend to reflect sleep-wake cycles rather than internal circadian rhythm. For a more complete assessment of sleep and activity levels, self-report questionnaires should be complemented by directly measured approaches such as actigraphy or polysomnography [61]. Use of electronic devices such as electronic light emitting e-books before bedtime has been shown to suppress melatonin secretion and delay DLMO [62]. To control the potential for such devices to impact outcomes, future studies may benefit from restricting light exposure, particularly to short wavelengths, during a defined period prior to sleep [32, 63–66]. Saliva samples analysed as part of the present study were collected at home, allowing participants to deposit their samples upon waking. No differences in sample collection times were observed between refractive groups. Participants could not be directly observed to ensure strict adherence to the collection protocol; however, the risk of deviation was mitigated through provision of detailed written instructions and video demonstration of the “passive drool” collection technique. Participants were also quizzed with respect to their adherence to the saliva collection protocol when they attended for data collection.

## 5. Conclusion

The elevated melatonin levels in adult myopes reported by Flanagan et al. were not observed in morning salivary samples provided by the adolescent cohort in the present study, despite comparable methodology, season of data collection, and geographical location. The adolescent participants in the present study demonstrated increased and more variable melatonin levels than previous adult studies, and it is not possible to determine from this cross-sectional study whether melatonin status prior to myopia onset is indicative of future refractive trajectory. Prospective studies are needed to determine whether melatonin levels during childhood are associated with an increased risk for faster myopia progression and/or higher future levels of myopia. Future studies exploring circadian factors should opt for a comprehensive DLMO protocol to determine circadian phase.

## Figures and Tables

**Table 1:** Sex and median (95% CI) age, SER, axial length, morning salivary melatonin concentration (pg/ml), PSQI scores, and PAC-Q scores for myopic and nonmyopic participants.

	Characteristic	All participants *n* = 70	Myopes *n* = 22	Nonmyopes *n* = 48	*P* value
Demographic	Sex, male/female, *n*	27/43	10/12	17/31	0.59^†^
	Age (years)	14.3 (14.2–14.5)	14.3 (14.2–14.7)	14.2 (14.1–14.7)	0.44

Ocular profile	SER (DS)	+0.22 (−0.19 to 0.5)	−2.16 (−3.81 to −1.31)	+0.60 (+0.44 to +0.75)	**<0.001 ** ^*∗*^
	SER range (DS)	−5.38 to +1.88	−5.38 to −0.69	−0.37 to +1.88	
	AXL (mm)	23.70 (23.42–24.09)	24.52 (23.95–25.18)	23.41 (23.12–23.70)	**<0.001 ** ^*∗*^

Melatonin profile	Melatonin conc/*n* (pg/ml)	4.82 (3.50–5.35)	4.52 (2.60–6.02)	4.89 (3.18–5.66)	0.91
	Time saliva sample collected	08:47 (08:30−09:00)	08:50 (08:20−09:00)	08:46 (08:30−09:00)	0.93

Sleep profile	Bedtime (hh:mm)	23:30 (23:00–23:50)	23:00 (22:59−00:00)	23:30 (23:00—00:00)	0.56
	Sleep latency (mins)	20 (15–30)	20 (10–30)	20 (10–30)	0.95
	Wake time (hh:mm)	08:59 (8:45−9:00)	08:52 (8:00–9:00)	09:00 (8:45−9:00)	0.88
	Time asleep (hh:mm)	08:30 (08:00–09:00)	09:00 (08.00—09.00)	08:15 (08:00—09.00)	0.26
	Global PSQI score	4.00 (4.00–5.00)	4.00 (3.00–6.00)	4.00 (3.00–5.00)	0.83
	Sleep quality (good/poor) (% poor)	22/70 (31%)	15/7 (32%)	33/15 (31%)	1.00

Activity profile	PAQ-C score	1.84 (1.50–2.27)	1.71 (1.30–2.31)	1.95 (1.48–2.46)	0.50^†^

SER = spherical equivalent refraction; AXL = axial length; PSQI = Pittsburgh Sleep Questionnaire Index; PAQ-C = Physical Activity Questionnaire for Children; CI = confidence interval; participants were labelled as having good sleep quality if PSQI score ≤5 and poor sleep quality if PSQI score >5 [28]; *P* values for Mann–Whitney *U* test (exception for ^†^which indicates chi-squared test). ^*∗*^indicates significance at *P* < 0.05.

**Table 2:** Relationship between age, SER, axial Length, time of saliva sample collection, bedtime, sleep latency, wake time, time asleep, global PSQI score, PAQ-C score, and morning salivary melatonin concentration.

Characteristic	Salivary melatonin	
	*ρ*	*P*
Age	0.075	0.54
SER	0.081	0.51
Axial length	0.090	0.46
Time saliva sample collected	0.060	0.62
Bedtime	−0.008	0.95
Sleep latency	0.021	0.86
Wake time	0.046	0.70
Time asleep	0.103	0.40
Global PSQI score	−0.003	0.98
PAQ-C score	−0.105	0.39

SER = spherical equivalent refraction; PSQI = Pittsburgh Sleep Questionnaire Index; PAQ-C = Physical Activity Questionnaire for Children; *ρ* = Spearman's correlation coefficient. ^*∗*^indicates significance at *P* < .05.

## Data Availability

The data used to support the findings of this study may be released upon application to the authors of the study, who can be contacted by e-mail at fulton-j6@ulster.ac.uk.

## Supplementary Materials

Supplementary Materials S1 Appendix. The bioanalytical method for salivary melatonin by liquid chromatography-tandem mass spectrometry. S2 STROBE Checklist. Checklist of items that should be included in reports of cross-sectional studies.
