# Supplementary material for: A Cross-Sectional Study of Myopia and Morning Melatonin Status in Northern Irish Adolescent Children
Source: J Ophthalmol. 2023 Nov 1;2023:7961623. doi: 10.1155/2023/7961623 (PMC10632006; doi:10.1155/2023/7961623)
Supplement: Supplementary Materials — S1 Appendix. The bioanalytical method for salivary melatonin by liquid chromatography-tandem mass spectrometry. S2 STROBE Checklist. Checklist of items that should be included in reports of cross-sectional studies. [file 7961623.f1.zip › S1 Appendix.pdf]

S1 Appendix.

Bioanalytical method for salivary melatonin by liquid chromatography-tandem mass spectrometry.

Chromatographic Conditions

Analytical column Inertsil ODS -EP 3.0 x 100 mm; 5um was purchased from GL Sciences (CA, US). Flow rate was set at 0.35 mL/min (constant), column temperature was set at 30°C, and injection volume was 50 µL. Sample diluent was 0.1% ascorbic acid with 0.001% ETDA in 50:50 (methanol/water, v/v). Mobile phases were (A) 70:30 (v/v) water: acetonitrile with 0.1% v/v of formic acid and (B) Acetonitrile with 0.1% v/v of formic acid.

Gradient Profile

Time (min)	Flow (ml/min)	%A	%B
0.0	0.35	100	0
5.0	0.35	100	0
10.0	0.35	40	60
10.1	0.35	100	0
15.1	0.35	100	0

Mass Spectrometer Conditions

Analysis was carried out using turbo ion spray source in positive ion mode and multiple reaction monitoring acquisition mode. Online tuning was performed to maximize parent isolation and product ion generation. Ionization and fragmentation conditions were set as follows: CAD (Arb): 6, CUR (Arb): 40, GSI (Arb): 35; GSII (Arb) 40; IS: (V) 5500; temp (°C): 500.

Multiple reaction monitoring transition table

Compound	Precursor (*m/z*)	Product *(m/z)*
**Melatonin**	233.1	174.2 (quant)
	233.1	159.0 (qual)
**Internal Standard (ISTD)**	237.1	178.2

Sample Preparation

600 µL of saliva, 20 µl internal standard working solution and 500 µL hexane were accurately pipetted into a 1.5 ml Eppendorf tube and vortexed at full speed for 30 seconds. Centrifugation was then performed for 5 min at 10 000 RPM at 4°C and the washed sample (bottom phase) was transferred to a fresh 1.5ml Eppendorf tube. 600 µL of dichloromethane and 100 µL of 1M sodium hydroxide solution were added to the sample and vortexed at full speed for 10 seconds. Centrifugation was then performed for 10 min at 10 000 RPM at 4°C and the top phase containing the saliva was discarded. The dichloromethane containing free melatonin was transferred to a fresh 1.5ml Eppendorf tube. Evaporation was performed to dryness using nitrogen and gentle heat (~40°C). Samples were reconstituted in 100 µL of 50:50 methanol/water solution and vortexed at full speed for 10 seconds. The reconstituted sample was then transferred to a 1.5ml HPLC vial insert (5mm x 30mm) for HPLC/MS analysis.
